# The Presence of Stone Moroko (*Pseudorasbora parva*) Drives Divergent Sediment Resistome Profiles in Chinese Mitten Crab (*Eriocheir sinensis*) Polyculture Pond

**DOI:** 10.3390/foods14213626

**Published:** 2025-10-24

**Authors:** Yiran Hou, Rui Jia, Linjun Zhou, Bing Li, Jian Zhu

**Affiliations:** 1 Wuxi Fisheries College, Nanjing Agricultural University, Wuxi 214081, China; houyr@ffrc.cn (Y.H.); jiar@ffrc.cn (R.J.); 2 Key Laboratory of Integrated Rice-Fish Farming Ecology, Ministry of Agriculture and Rural Affairs, Freshwater Fisheries Research Center, Chinese Academy of Fishery Sciences, Wuxi 214081, China; zhoulinjun@ffrc.cn

**Keywords:** stone moroko (*Pseudorasbora parva*), Chinese mitten crab (*Eriocheir sinensis*) polyculture, metagenomics, antibiotic resistance genes, aquaculture sediment

## Abstract

The propagation of antibiotic resistance genes (ARGs) in aquatic environments poses a significant threat to global health. This study compared sediment resistome profiles in river crab (*Eriocheir sinensis*) polyculture systems with and without stone moroko (*Pseudorasbora parva*). The results showed that, compared to the control group (MC group), the sediment from the polyculture system containing stone moroko (PC group) exhibited significant reductions in the total abundances of ARGs, metal resistance genes (MRGs), biocide resistance genes (BRGs), and mobile genetic elements (MGEs). Crucially, the total abundance and composition of MGEs in pond sediment were substantially correlated with ARGs, MRGs, and BRGs, respectively. Co-occurrence network analysis revealed that there was only one edge between ARGs and MGEs in the PC group, whereas the MC group had eight edges. Additionally, the proportion of mobile ARGs in the PC group was significantly lower than that in the MC group. Alterations in resistome profiles were markedly associated with decreased levels of total carbon (TC) and phosphate in the sediment. All of the findings demonstrated that the introduction of stone moroko in the river crab polyculture system effectively mitigated the sediment resistome primarily by altering environmental factors and suppressing MGEs, thereby disrupting the horizontal transfer network of resistance genes. This study highlights the potential of leveraging aquatic biota as a novel biological strategy for the in situ management of environmental antimicrobial resistance.

## 1. Introduction

Antibiotic resistance has become a major global concern [1]. Antibiotic resistance genes (ARGs), as a novel type of environmental pollutant, are widespread in microbial communities through horizontal gene transfer (HGT), promoting the emergence of multidrug-resistant pathogens and posing a potential threat to global human health [2,3,4,5]. With the increasing global demand for aquatic products and the rapid development of aquaculture, the industry has become a significant source of antibiotic use [6,7]. During aquaculture processes, most antibiotics remain in the water body or pond sediment without being absorbed or metabolized, creating selective pressure that accelerates ARG generation and propagates ARGs via HGT mediated by mobile genetic elements (MGEs) such as plasmids, integrons, and transposons [6,8,9,10]. In addition to the selective pressure from the antibiotics themselves, antibacterial agents such as biocides and metals may also promote the development and dissemination of resistance to biocides, metals, and antibiotics through co-selection mechanisms, potentially leading to the emergence of multidrug-resistant bacteria [5,11]. Therefore, it is crucial to monitor the dynamic distribution of ARGs, metal resistance genes (MRGs), biocide resistance genes (BRGs), and MGEs in various environmental contexts to mitigate the spread of ARGs [5,12].

The ARGs are prevalent in aquaculture environments, particularly within sediments that act as reservoirs for both ARGs and dormant antibiotic-resistant bacteria (ARB), posing a high risk of antibiotic resistance and playing a critical role in the dissemination of ARGs [13,14,15]. Previous studies have demonstrated that drug use during aquaculture leads to the enrichment of ARGs in sediments, with these genes being consistently detectable even at minimal antibiotic concentrations and exhibiting notably greater abundance compared to sediments outside aquaculture ponds [16]. Even resistance genes for antibiotics that had never been used in some aquaculture farms were observed to be enriched in the sediments [17]. Antibiotic resistance in aquaculture environments can be potentially impacted by different farming practices and management methods, which could alter resistome profiles by selecting for resistance traits, modifying microbial community structures, or affecting the physicochemical properties of sediments [18,19,20]. Additionally, emerging evidence has revealed the consistent co-occurrence patterns and the co-selection processes between ARGs and MRGs within microbial genomes across diverse sediment matrices [19,21,22]. Given these considerations, comprehensive characterization of ARGs and their associated MRGs, BRGs, and MGEs in aquaculture sediment is essential for accurate human health risk assessment.

Promoting more sustainable aquaculture production patterns and management practices has emerged as a critical global priority [23,24]. Integrated aquaculture, centered on multispecies polyculture, has been proven to be more effective than traditional single-species aquaculture, significantly enhancing resource utilization efficiency and overall productivity while reducing environmental impact through the establishment of mutualistic interactions among species [25,26]. The Chinese mitten crab (*Eriocheir sinensis*), as the world’s third largest crustacean in terms of production, is an economically significant species of global importance and an excellent source of minerals and high-quality protein [27,28]. In 2023, the production of river crabs in China reached 888,629 t, accounting for approximately 16.7% of the total freshwater crustacean production in the country [29,30,31,32]. To improve both productivity and environmental sustainability, this species is commonly co-cultured with silver carp (*Hypophthalmichthys molitrix*) and mandarin fish (*Siniperca chuatsi*) [33,34]. In recent years, stone moroko (*Pseudorasbora parva*) has been experimentally introduced into this polyculture system [35,36]. The stone moroko is a small omnivorous fish feeding on algae, plankton, and organic detritus, and can significantly enhance the diversity and stability of aquatic microbial communities [36]. Changes in microbial community diversity inevitably affect the characteristics of the resistome in the environment [20]. However, the effects of such polyculture systems on the regulation of ARGs in the surrounding environment remain poorly understood.

This study systematically compared two river crab polyculture systems—with versus without stone moroko—through assessing the ARG, MRG, BRG, and MGE profiles. The research objectives were to: (1) evaluate the impacts of stone moroko on sediment resistome profiles, (2) identify key environmental drivers shaping the microbial resistomes in pond sediment, and (3) elucidate the interrelationships among ARGs, MRGs, BRGs, and MGEs in pond sediment. These findings will provide critical insights for developing mitigation strategies against antimicrobial resistance and advancing sustainable aquaculture practices.

## 2. Materials and Methods

### 2.1. Experimental Site and Design

**Figure 1:**
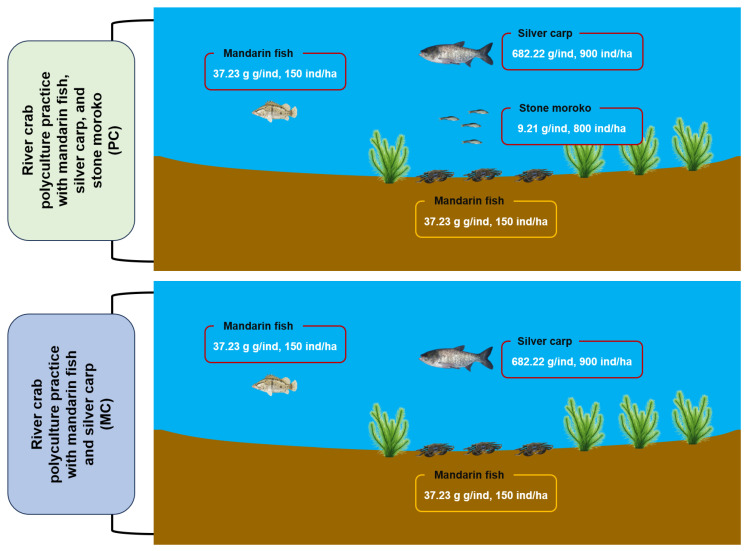
The schematic diagram of the conducted experiment.

The study was conducted at Changcheng River Crab Farm (119.82° E, 31.99° N) in Changzhou, Jiangsu Province, China (119.82° E, 31.99° N), utilizing two standardized 0.87-ha aquaculture ponds from 15 March to 15 October 2023, to compare two polyculture practices for Chinese mitten crab (*Eriocheir sinensis*) (Figure 1). The first polyculture practice (MC) involved co-culturing Chinese mitten crab with mandarin fish (*Siniperca chuatsi*) and silver carp (*Hypophthalmichthys molitrix*), while the second polyculture practice (PC) introduced an additional species, stone moroko (*Pseudorasbora parva*) (Figure 1). The initial stocking densities and average weights for various species were as follows: per hectare, 21,000 Chinese mitten crabs averaging 15.63 g each, 150 mandarin fish averaging 37.23 g each, 900 silver carps averaging 682.22 g each, and 800 stone moroko averaging 9.21 g each. All polyculture practices received daily feed comprising commercial pellets (Xinbei Menghe Hongzhiyu Aquaculture Farm, Changzhou, China), ice-fresh fish, and corn in a 1:7:2 ratio, administered at 3% of estimated crab biomass with weekly quantity adjustments. Water management involved biweekly exchanges replacing 20% of pond volume, maintaining consistent operational protocols between different polyculture practices.

Sampling was conducted on 3 September 2023 during the peak culture period of Chinese mitten crabs. Ten sampling points were set up using a systematic sampling method in each pond, with ten sediment subsamples collected per point and homogenized into one composite sample, yielding ten replicates per treatment group (PC and MC). The sediment samples were divided for dual analyses: one portion for quantifying total carbon (TC), total nitrogen (TN), total phosphorus (TP), total sulfur (TS), ammonia, nitrate, nitrite, and phosphate; the other for DNA extraction and metagenomic sequencing.

### 2.2. Sediment Properties Determination

Prior to physicochemical analysis, sediment samples underwent freeze-drying at −50 °C for 72 h using a Haier Biomedical freeze-dryer (Haier, Qingdao, China). The lyophilized samples were then ground with a mortar and sieved through a 100-mesh screen. The TC, TN, and TS concentrations were quantified using a Vario EL Cube elemental analyzer (Elementar, Frankfurt, Germany). The sediment TP content was determined through alkali fusion–Mo-Sb anti spectrophotometric method [37]. Ammonia, nitrite, and nitrate in sediment were extracted with 2 mol/L KCl solution and then measured, respectively by Nessler’s reagent spectrophotometry, Diazo coupling spectrophotometry, and ultraviolet spectrophotometry methods [38,39].

### 2.3. DNA Extraction and Metagenomic Sequencing

Microbial DNA from pond sediment samples was extracted using the FastDNA Spin Kit for Soil (MP Biomedicals, Irvine, CA, USA), and following verification via agarose gel electrophoresis (1.5%) and spectrophotometry (NanoDrop 1000, Thermo Fisher Scientific Inc., Waltham, MA, USA), libraries were prepared for sequencing using the NEBNext^®^ Ultra™ DNA Library Prep Kit for Illumina (New England Biolabs, Ipswick, MA, USA). DNA fragments of 350 bp were obtained by ultrasonication, followed by end repair, A-tailing, and ligation of Illumina sequencing adapters. After PCR amplification, the libraries were purified using the AMPure XP system (Beckman Coulter, Coulter, IA, USA) and assessed for quality with an Agilent 2100 Bioanalyzer (Agilent Technologies, Santa Clara, CA, USA). Metagenomic sequencing was carried out on the Illumina NovaSeq 6000 platform, with sterile water controls used throughout the experiment. Raw data were quality controlled with the NGS QC Toolkit to remove low-quality sequences (Phred < 30, ambiguous bases, length < 150 bp), resulting in clean data for subsequent analysis [40].

### 2.4. Microbial Resistome Annotations

The BLASTX v2.15.0 was used to compare functional genes against the SARG v3.0-M, MobileGeneticElement, and BacMet2 databases to identify and annotate ARGs, MGEs, MRGs, and BRGs. The alignment parameters were set to an e-value of ≤10^−10^, similarity ≥ 80%, and query coverage ≥ 70% [41,42]. The metagenomic datasets in the pond sediment samples were analyzed using the arg_ranker v3.0 framework to assess the risk to human health posed by the resistome in sediment samples [43]. Within this framework, ARGs are categorized into four risk levels based on their mobility, host pathogenicity, and enrichment in human-associated environments. Rank I contains the highest risk ARGs, whereas rank IV contains the lowest risk ARGs [20,44]. The resistome risk index was determined by the results from arg_ranker v3.0 including the percentage and total abundance of each rank and the rank risk code [45].

### 2.5. Bioinformatics Analysis and Statistics

All statistical analyses were performed on the R v4.2.2 platform, and results were visualized using the “ggplot2” package v4.0.0. Differences in all indicators related to sediment resistome characteristics, resistome risk, and environmental factors between the PC and MC groups were compared using the Wilcoxon rank-sum test. The Bray–Curtis distances for the ARGs, MGEs, MRGs, and BRGs were calculated, and the overall differences in the resistome profiles within pond sediment between the PC and MC groups were assessed using principal coordinates analysis (PCoA) accompanied by the adonis test. Linear regression analysis, mantel test, and procrustes analysis were employed to clarify the correlations between the MGEs and the ARGs, MRGs, and BRGs, respectively. Random forest analysis was used to identify the contribution of different MGE types to the variations in the characteristics of ARGs, MRGs, and BRGs. Based on Spearman correlation matrices (Spearman’s r > 0.8 and *p*-value < 0.05), co-occurrence networks were constructed to reveal the interaction patterns between ARGs, MRGs, BRGs, and MGEs in the MC and PC groups, respectively. Mantel tests and distance-based redundancy analysis (db-RDA) were applied to evaluate the correlations between environmental variables and the ARGs, MRGs, and BRGs. The importance of different environmental factors to the variations in ARGs, MRGs, BRGs, and MGEs were also identified using random forest analysis.

## 3. Results

### 3.1. Resistome Profiles in Pond Sediment

**Figure 2:**
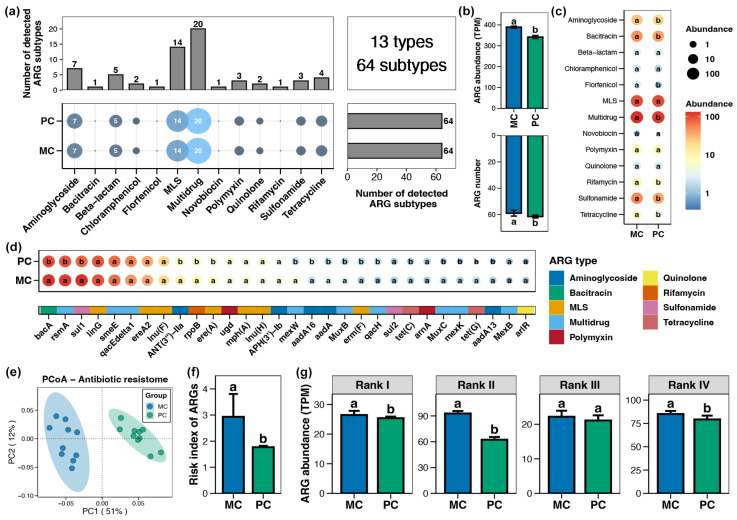
Characteristics of the antibiotics resistance genes (ARGs) in pond sediment. (**a**) Numbers of the detected ARG types and subtypes in pond sediment samples. (**b**) Differences in the number and abundance of the sediment ARGs between PC and MC groups. (**c**) Differences in the abundances of the ARG types in pond sediment between PC and MC groups. (**d**) Differences in the abundances of the dominant ARG subtypes (top 30 by relative abundance) in pond sediment between PC and MC groups. (**e**) Principal co-ordinates analysis (PCoA) based on Bray–Curtis distances revealing the difference in the ARGs profiles between PC and MC groups. (**f**) Differences in the risk index of ARGs in pond sediment between the PC and MC groups. (**g**) Differences in the abundances of ARGs ranked into four levels based on their potential threat to human health between the PC and MC groups. Significant differences (*p* < 0.05) are indicated by non-matching superscript letters throughout.

A total of 13 types and 64 subtypes of ARGs were detected in the pond sediment samples, with multidrug resistance genes being the most abundant, followed by macrolide-lincosamide-streptogramin (MLS) resistance genes (Figure 2a). Both MC and PC group sediments contained all 64 ARG subtypes (Figure 2a). However, the total abundance of ARGs within pond sediment in the PC group was significantly lower than that in the MC group (Figure 2b, Wilcoxon rank-sum test, *p* < 0.05). At the type and subtype (top 30 by relative abundance) levels, 7 and 17 significantly different ARGs were identified in the pond sediment between the PC and MC groups, respectively. All differential ARGs showed significantly decreased abundances in the PC group, with no ARGs demonstrating increased abundance in the PC group (Figure 2c,d, Wilcoxon rank-sum test, *p* < 0.05). Specifically, the abundances of multidrug, MLS, sulfonamide, bacitracin, and aminoglycoside resistance genes in the PC group were notably lower than those in the MC group at the type level (Figure 2c, Wilcoxon rank-sum test, *p* < 0.05). PCoA analysis also revealed distinct separation in the sediment ARGs profiles between the MC and PC groups (Figure 2e, Adonis test, *p* < 0.05). When ARGs were categorized into four risk ranks and the risk index of ARGs in the samples was calculated, the results revealed that the PC group considerably reduced the risk index of ARGs in pond sediment, with this reduction primarily attributed to the decreased abundance of rank II ARGs (Figure 2f,g, Wilcoxon rank-sum test, *p* < 0.05).

**Figure 3:**
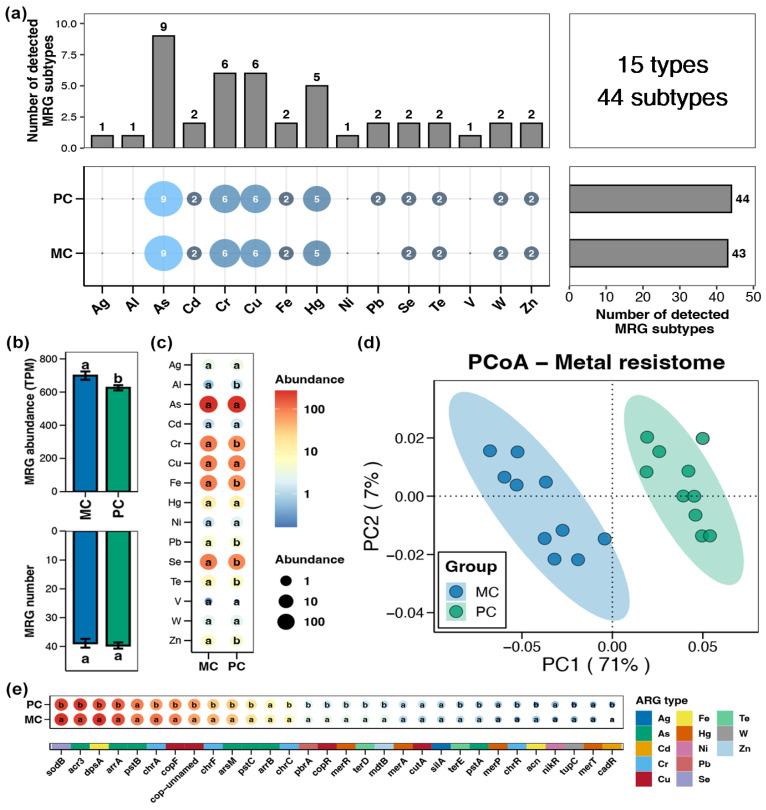
Characteristics of the metal resistance genes (MRGs) in pond sediment. (**a**) Numbers of the detected MRG types and subtypes in the pond sediment samples. (**b**) Differences in the number and abundance of the sediment MRGs between the PC and MC groups. (**c**) Differences in the abundances of the MRG types in pond sediment between the PC and MC groups. (**d**) Principal co-ordinates analysis (PCoA) based on Bray–Curtis distances revealing the difference in the MRGs profiles between the PC and MC groups. (**e**) Differences in the abundances of the dominant MRG subtypes (top 30 by relative abundance) in pond sediment between the PC and MC groups. Significant differences (*p* < 0.05) are indicated by non-matching superscript letters throughout.

A total of 15 types and 44 subtypes of MRGs were detected in the pond sediment samples, with the As resistance gene exhibiting the highest subtype number, followed by the Cr, Cu, and Hg resistance genes (Figure 3a). The total abundance of sediment MRGs in the PC group was significantly lower than that in the MC group (Figure 3b, Wilcoxon rank-sum test, *p* < 0.05). At the type and subtype (top 30 by relative abundance) levels, 7 types and 22 subtypes of MRGs showing obvious differences between the PC and MC groups were identified, respectively. Similarly, all the differential MRGs exhibited significantly reduced abundances in the PC group, with no differential MRGs found to increase in abundance in the PC group (Figure 3c,e, Wilcoxon rank-sum test, *p* < 0.05). Specifically, at the type level, the abundances of Al, Cr, Fe, Pb, Se, Te, and Zn resistance genes within pond sediment in the PC group were substantially lower than those in the MC group (Figure 3c, Wilcoxon rank-sum test, *p* < 0.05). In the PCoA analysis, the sediment MRGs of the MC and PC groups clustered separately, indicating distinct differences in the MRG profiles within pond sediment between the MC and PC groups (Figure 3d, Adonis test, *p* < 0.05).

**Figure 4:**
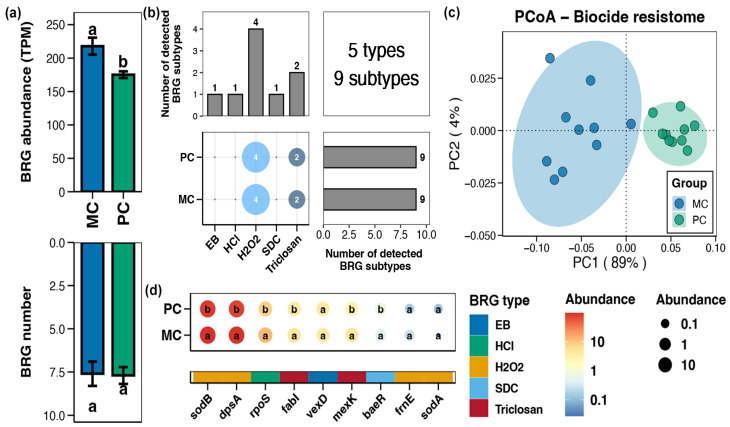
Characteristics of the biocide resistance genes (BRGs) in pond sediment. (**a**) Differences in the number and abundance of the sediment BRGs between the PC and MC groups. (**b**) Numbers of the detected BRG types and subtypes in the pond sediment samples. (**c**) Principal co-ordinates analysis (PCoA) based on Bray–Curtis distances revealing the difference in the BRGs profiles between the PC and MC groups. (**d**) Differences in the abundances of the MRG subtypes in pond sediment between the PC and MC groups. Significant differences (*p* < 0.05) are indicated by non-matching superscript letters throughout.

In the pond sediment, a total of 5 types and 9 subtypes of BRGs were detected (Figure 4b). Compared to the MC group, the total abundance of sediment BRGs in the PC group substantially decreased (Figure 4a, Wilcoxon rank-sum test, *p* < 0.05). In the PCoA analysis, the BRGs from the MC and PC groups clustered separately, indicating clear differences in the sediment BRGs characteristics between the MC and PC groups (Figure 4c, Adonis test, *p* < 0.05). The abundances of the BRGs subtypes within pond sediment including *sodB*, *dpsA*, *rpoS*, *fabI*, *mexK*, and *baeR* were considerably lower than in the MC group (Figure 4d, Wilcoxon rank-sum test, *p* < 0.05).

**Figure 5:**
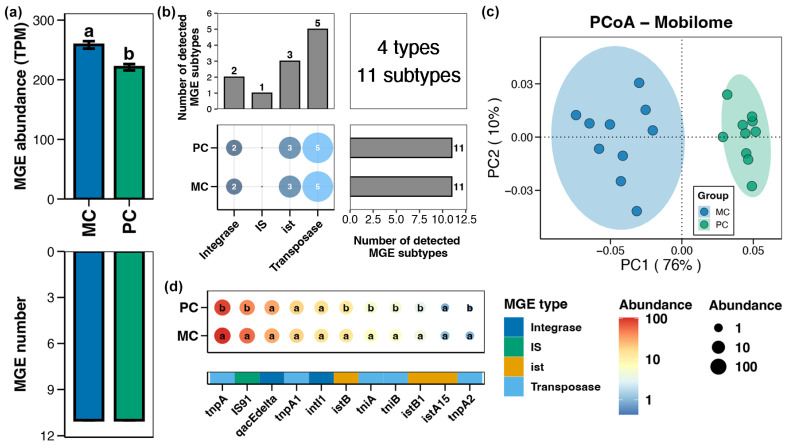
Characteristics of the mobile genetic elements (MGEs) in pond sediment. (**a**) Differences in the number and abundance of the sediment MGEs between the PC and MC groups. (**b**) Numbers of the detected MGE types and subtypes in pond sediment samples. (**c**) Principal co-ordinates analysis (PCoA) based on Bray–Curtis distances revealing the difference in the MGEs profiles between the PC and MC groups. (**d**) Differences in the abundances of the MGE subtypes in pond sediment between the PC and MC groups. Significant differences (*p* < 0.05) are indicated by non-matching superscript letters throughout.

A total of 4 types and 11 subtypes of MGEs were identified in the pond sediment samples (Figure 5b). Compared to the MC group, the total abundance of MGEs in the pond sediment of the PC group was remarkably reduced (Figure 5a, Wilcoxon rank-sum test, *p* < 0.05). PCoA analysis exhibited distinct clusters in the sediment MGEs between the MC and PC groups, suggesting significant differences in the MGE profiles between the two groups (Figure 5c, Adonis test, *p* < 0.05). Specifically, the abundances of sediment MGEs such as *tnpA*, *IS91*, *istB*, *tniA*, *tniB*, *istB1*, and *tnpA2* were significantly lower in the PC group compared to the MC group (Figure 5d, Wilcoxon rank-sum test, *p* < 0.05).

### 3.2. Correlations Between MGEs and Resistance Genes in Pond Sediment

**Figure 6:**
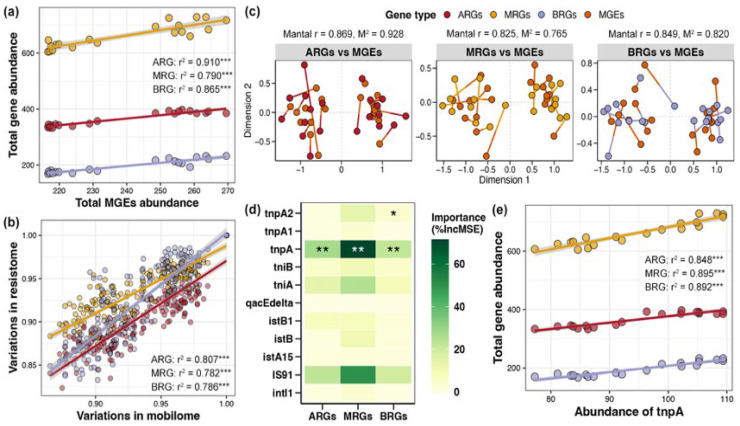
Correlations between mobile genetic elements (MGEs) and resistance genes in pond sediment. (**a**) Linear regression analysis evaluating the correlations between the total abundance of MGEs and the total abundances of antibiotic resistance genes (ARGs), metal resistance genes (MRGs), and biocide resistance genes (BRGs), respectively. The linear regression results are depicted with red, yellow, and purple lines for the ARGs, MRGs, and BRGs, respectively. (**b**) Linear regression analysis assessing the correlations between the MGEs composition and the ARGs, MRGs, and BRGs compositions, respectively. Red, yellow, and purple lines represent the linear regression results of the ARGs, MRGs, and BRGs with MGEs, respectively. (**c**) Mantel test and Procrustes analysis characterizing the relationships between the MGE composition and the ARG, MRG, and BRG compositions, respectively. (**d**) Random forest analysis identifying the importances of different MGEs on the ARGs, MRGs, and BRGs. Asterisks “*”, “**” and “***” indicate significant effects for each specific MGE, with significance levels of *p* < 0.05, *p* < 0.01, and *p* < 0.001, respectively. (**e**) Linear regression analysis evaluating the correlations between the abundance of the *tnpA* gene and the total abundances of ARGs, MRGs, and BRGs, respectively. The linear regression results are depicted with red, yellow, and purple lines for ARGs, MRGs, and BRGs, respectively.

Linear regression revealed obvious linear relationships between the total abundance of MGEs in the pond sediment and the total abundances of ARGs, MRGs, and BRGs, respectively, with the association strength ranking as ARG (r^2^ = 0.910) > BRG (r^2^ = 0.865) > MRG (r^2^ = 0.790) (Figure 6a, *p* < 0.05). Simultaneously, based on the Bray–Curtis distance, there were significant distance-dependent similarities between the composition of MGEs and the compositions of ARGs, MRGs, and BRGs, respectively, with the association strength ranking as ARG (r^2^ = 0.807) > BRG (r^2^ = 0.786) > MRG (r^2^ = 0.782) (Figure 6b, *p* < 0.05). Mantel tests and Procrustes analysis further indicated remarkable associations between the composition of MGEs and the compositions of ARGs, MRGs, and BRGs, respectively, again with the association strength ARG (r^2^ = 0.869) > BRG (r^2^ = 0.849) > MRG (r^2^ = 0.825) (Figure 6c, *p* < 0.05). Random forest analysis identified the key MGE markedly influencing the ARGs, MRGs, and BRGs as gene *tnpA*, which could be used as an indicator gene to gauge the total abundance of ARGs, MRGs, and BRGs in pond sediment (Figure 6d, *p* < 0.05). Further linear regression evaluated the considerable correlations of *tnpA* gene abundance with the total abundances of ARGs, MRGs, and BRGs, respectively (Figure 6e, *p* < 0.05).

**Figure 7:**
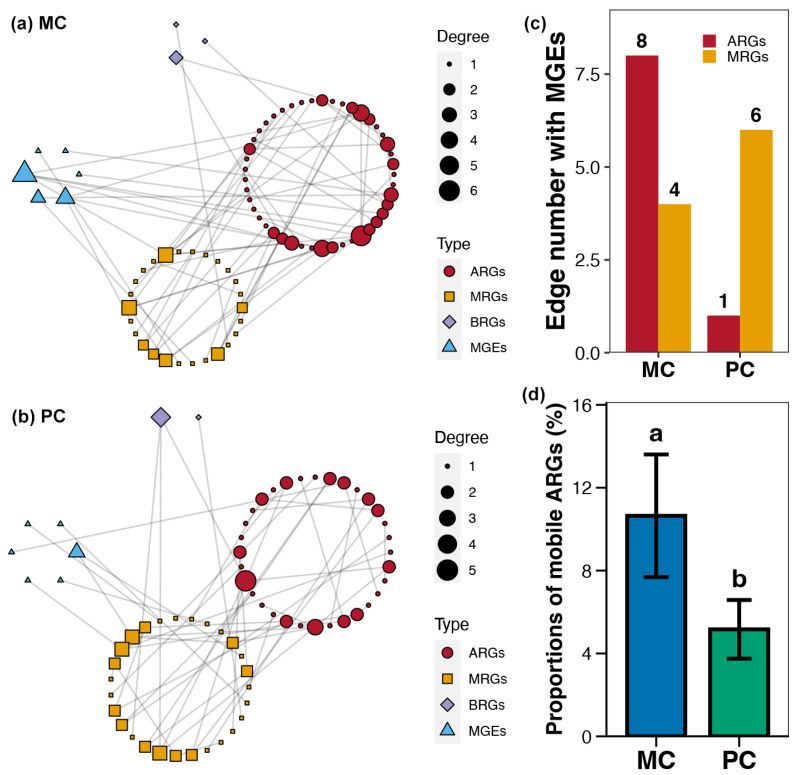
Co-occurrence networks for the sediment resistome in different polyculture practices. (**a**) Co-occurrence networks for the antibiotic resistance genes (ARGs), metal resistance genes (MRGs), biocide resistance genes (BRGs), and mobile genetic elements (MGEs) within pond sediment in the MC group. (**b**) Co-occurrence networks for the ARGs, MRGs, BRGs, and MGEs within pond sediment in the PC group. (**c**) Differences in the number of edges between MEGs and ARGs or MRGs across the PC and MC groups. (**d**) Difference in the proportions of mobile ARGs in pond sediment between the PC and MC groups. Significant differences (*p* < 0.05) are indicated by non-matching superscript letters throughout.

Using co-occurrence network analysis, the association patterns between ARGs, MRGs, BRGs, and MGEs within pond sediment in the MC and PC groups were obtained (Figure 7a,b). It could be seen that the overall degree and number of nodes in the co-occurrence network for the PC group were lower than those for the MC group (Figure 7a,b). The number of ARG-MGE associations (edges) within the co-occurrence network in the PC group was noticeably lower than in the MC group (Figure 7c). Additionally, the proportion of mobile ARGs in the PC group was significantly lower than in the MC group (Figure 7c).

### 3.3. Correlations Between Environmental Variables and Resistome in Pond Sediment

**Figure 8:**
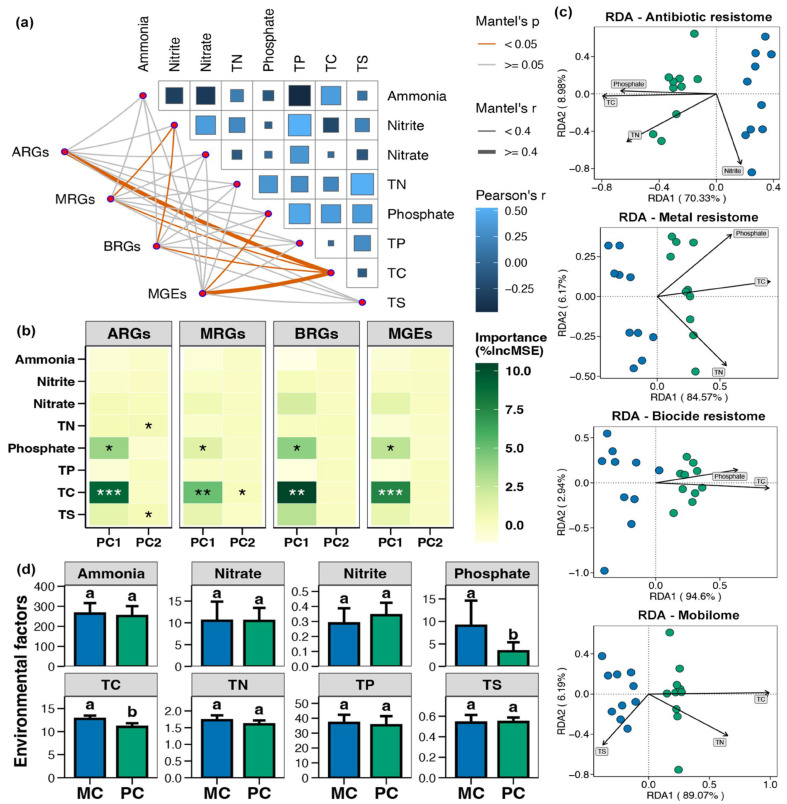
Correlations between environmental variables and resistome in pond sediment. (**a**) Mantel test assessing the correlations between the environmental variables and the antibiotic resistance genes (ARGs), metal resistance genes (MRGs), and biocide resistance genes (BRGs), respectively. TN, TP, TC, and TS represent the total nitrogen, total phosphorus, total carbon, and total sulfur, respectively. (**b**) Random forest analysis identifying the relative contributions of different environmental factors on the variations in the ARGs, MRGs, BRGs, and MGEs. Asterisks “*”, “**”, and “***” indicate significant impact for the environmental factor, with significance levels of *p* < 0.05, *p* < 0.01, and *p* < 0.001, respectively. (**c**) Distance-based redundancy analysis (db-RDA) exhibiting correlations of the sediment parameters with the ARGs, MRGs, BRGs, and MGEs in pond sediment, respectively. (**d**) Differences in the total carbon (TC, μg/mg), total nitrogen (TN, μg/mg), total phosphorus (TP, μg/g), total sulfur (TS, μg/mg), ammonia (μg/g), nitrate (μg/g), nitrite (μg/g), and phosphate (μg/g) concentrations within pond sediment between the PC and MC groups. Significant differences between the PC and MC groups for corresponding indicators are denoted by different lowercase letters, while shared letters indicate no statistically significant difference.

The Mantel test indicated that the ARGs, MRGs, BRGs, and MGEs in pond sediment were considerably correlated with the TC level, and both MRGs and BRGs were also significantly correlated with the nitrite content (Figure 8a, *p* < 0.05). Random forest analysis suggested that the TC, TN, TS, and phosphate contents were likely key factors driving the variations in sediment resistome (Figure 8b, *p* < 0.05). RDA analysis demonstrated that in pond sediment, both ARGs and MRGs were substantially correlated with the TC, TN, and phosphate levels, whereas BRGs were remarkably correlated with the TC and phosphate content (Figure 8c, *p* < 0.05). MGEs were significantly correlated with the TC, TN, and TS contents (Figure 8c, *p* < 0.05). The TC and phosphate concentrations in pond sediment were significantly decreased in the PC group compared with the MC group (Supplementary Materials, Figure 8d, Wilcoxon rank-sum test, *p* < 0.05).

## 4. Discussion

Our results demonstrated that the presence of stone moroko induced a significant shift in the genetic landscape of pond sediment, markedly reducing the abundance and altering the composition of the ARGs, MRGs, BRGs, and, most critically, MGEs in pond sediments compared to the MC group. The most pivotal finding of this study was the remarkable parallel reduction in MGEs alongside ARGs, MRGs, and BRGs, with a notable correlation observed between the abundance of MGEs and that of ARGs, MRGs, and BRGs. Reduction in MGEs was of paramount importance because MGEs are the fundamental engines of horizontal gene transfer (HGT), facilitating the dissemination and enrichment of resistance genes across microbial populations [46,47]. Previous studies have indicated that among the MGEs, *tnpA* possesses the greatest potential for spreading ARGs and exhibits the highest co-occurrence frequency with multiple ARGs [48,49]. Correspondingly, our random forest model identified *tnpA* as the single most important MGE affecting the resistome in pond sediment. Therefore, the main mechanism by which stone moroko mitigated the sediment resistome in this study was likely through the suppression of MGE activity, especially that of *tnpA*-associated transposons, thereby effectively disrupting the HGT network responsible for propagating resistance traits.

Notably, not a single ARG, MRG, or BRG subtype was found to increase in abundance, and all statistically different subtypes exhibited substantial decreases. This pattern suggests that the impact of stone moroko is broad-spectrum, non-selective, and ultimately mitigates the reservoir of resistance genes in the sediment environment. The suppression of *tnpA* and other MGEs could provide a unified explanation for the observed co-reduction in ARGs, MRGs, and BRGs. Genetic determinants for resistance to antibiotics, metals, and biocides are frequently found on the same mobile genetic elements, such as plasmids and transposons [50,51,52]. Emerging evidence has revealed the consistent co-occurrence patterns and the co-selection processes between ARGs, MRGs, and BRGs within microbial genomes across animal feces, intestines, and diverse sediment matrices [19,21,22,52,53,54]. The suppression of MGEs, particularly the transposase gene *tnpA* which facilitated the movement of these platforms, would therefore result in a coordinated reduction across all co-located resistance types, which was precisely what we observed. This mechanism was also vividly illustrated by the co-occurrence network analysis. The simplified network topology, reduced number of edges, and, specifically, the fewer connections between ARGs and MGEs in the PC group depicted a disrupted HGT network. The considerable decrease in the proportion of mobile ARGs further confirmed that the introduction of stone moroko not only reduced the abundance of resistance genes, but also critically diminished their potential for dissemination. As revealed by the arg_ranker v3.0 framework, this reduction effectively mitigated the ecological risk posed by ARGs and their associated threats to human health [43].

Multiple studies have shown that changes in environmental factors may affect the abundance of resistance genes and their horizontal transfer [55,56,57,58]. The present study identified the environmental drivers that were likely modulated by stone moroko and that facilitated this process. Compared to the MC group, stone moroko presence markedly decreased the TC and phosphate concentrations in pond sediment. In addition, random forest and RDA analyses further confirmed that TC and phosphate were key factors shaping the sediment resistome. These findings were strongly supported by prior research. As Wanyan et al. [59] demonstrated, high-risk ARGs (e.g., *floR*) exhibited positive correlations with TP and TC, suggesting that nutrient enrichment provides a favorable niche for the persistence and proliferation of resistant bacteria. This relationship is further corroborated by studies across diverse aquatic environments [60,61]. For instance, even in a marine aquaculture ecosystem, a significant positive correlation between total organic carbon and predominant ARGs has been observed [61]. Excessive phosphorus input also exacerbates the dissemination of antibiotic resistance genes in soil and aquatic ecosystems [62,63]. Therefore, the introduction of stone moroko in the PC group likely influenced the resistome in pond sediment by reducing the TC and phosphate levels. Meanwhile, nutrients play a critical role in regulating bacterial growth, abundance, and metabolic activity, thereby exerting a strong influence on the structure and composition of bacterial communities [64,65]. We propose a possible direct mechanism: the introduction of stone moroko reduced the availability of TC and phosphate in the sediment, thereby diminishing the environmental conditions that support and enrich MGEs and their associated resistance genes. However, this still requires further research for verification.

## 5. Conclusions

This study provides compelling evidence that the introduction of stone moroko in a river crab polyculture system induced a marked and comprehensive reduction in the sediment resistome, encompassing ARGs, MRGs, and BRGs. The central finding of this work was that this mitigation was achieved primarily through the suppression of MGEs, the key engines for horizontal gene transfer. The transposase gene *tnpA* was identified as the most critical indicator, with its abundance serving as an accurate predictor for the overall burden of resistance genes in the sediment. In addition, the stone moroko presence altered the sediment microenvironment, leading to significant reductions in the TC and phosphate levels, which were identified as primary drivers for the alterations in resistome profiles.

In summary, we proposed a possible mechanism: the introduction of stone moroko reduced sediment the TC and phosphate levels, which in turn suppressed the abundance and mobilization potential of MGEs like *tnpA*. This ultimately led to a collapse in the reservoir of mobile ARGs, MRGs, and BRGs. Our findings suggest that manipulating aquatic biota could be a promising, eco-friendly strategy for the in-situ remediation of contaminated sediments and the mitigation of environmental antimicrobial resistance risks. Future research should focus on elucidating the precise bioturbation processes that lead to nutrient reduction and exploring the application of this concept in different aquatic ecosystems. However, it should be noted that this study focused on key nutrient parameters; future work incorporating a broader range of sediment properties, such as pH and organic matter quality, would provide a more holistic understanding of the resistome dynamics.

## Data Availability

The raw data supporting the conclusions of this study are available from the corresponding author upon reasonable request.

## Supplementary Materials

The following supporting information can be downloaded at: https://www.mdpi.com/article/10.3390/foods14213626/s1, Table S1: Differences in the total carbon (TC, μg/mg), total nitrogen (TN, μg/mg), total phosphorus (TP, μg/g), total sulfur (TS, μg/mg), ammonia (μg/g), nitrate (μg/g), nitrite (μg/g), and phosphate (μg/g) concentrations within pond sediment between the PC and MC groups.

## Abbreviations

The following abbreviations are used in this manuscript:

ARG	Antibiotic resistance genes
MRG	Metal resistance genes
BRG	Biocide resistance genes
MGE	Mobile genetic elements
HGT	Horizontal gene transfer
TC	Total carbon
TN	Total nitrogen
TP	Total phosphorus
