# Supplementary material for: The Presence of Stone Moroko (Pseudorasbora parva) Drives Divergent Sediment Resistome Profiles in Chinese Mitten Crab (Eriocheir sinensis) Polyculture Pond
Source: Foods. 2025 Oct 24;14(21):3626. doi: 10.3390/foods14213626 (PMC12608861; doi:10.3390/foods14213626)
Supplement: Supplementary file 1 [file foods-14-03626-s001.zip › foods-3906473-supplementary.docx]

**Supplementary Materials:**

The presence of stone moroko (*Pseudorasbora parva*) drives divergent sediment resistome profiles in Chinese mitten crab (*Eriocheir sinensis*) polyculture pond

Table S1. Differences in the total carbon (TC, μg/mg), total nitrogen (TN, μg/mg), total phosphorus (TP, μg/g), total sulfur (TS, μg/mg), ammonia (μg/g), nitrate (μg/g), nitrite (μg/g), and phosphate (μg/g) concentrations within pond sediment between the PC and MC groups.

| Groups | TC | TN | TP | TS | Ammonia | Nitrate | Nitrite | Phosphate |
| --- | --- | --- | --- | --- | --- | --- | --- | --- |
| PC | 11.02±0.78b | 1.60±0.12a | 35.26±6.09 | 0.54±0.04 | 251.31±49.20a | 10.44±2.99a | 0.34±0.08a | 3.37±1.19b |
| MC | 12.75±0.72a | 1.72±0.15a | 36.82±5.49 | 0.54±0.07 | 263.51±52.50a | 10.47±4.39a | 0.29±0.10a | 9.04±5.58a |

Significant differences between PC and MC groups for corresponding indicators are denoted by different lowercase letters, while shared letters indicate no statistically significant difference.
